# Reference genome-independent assessment of mutation density using restriction enzyme-phased sequencing

**DOI:** 10.1186/1471-2164-13-72

**Published:** 2012-02-14

**Authors:** Jennifer Monson-Miller, Diana C Sanchez-Mendez, Joseph Fass, Isabelle M Henry, Thomas H Tai, Luca Comai

**Affiliations:** 1 Department of Plant Biology and Genome Center, UC Davis, Davis, California 95616, USA; 2 Crops Pathology and Genetics Research Unit, U.S. Department of Agriculture, Agricultural Research Service, Davis, California 95616, USA; 3 Bioinformatics Core, Genome Center, UC Davis, Davis, California 95616, USA

## Abstract

**Background:**

The availability of low cost sequencing has spurred its application to discovery and typing of variation, including variation induced by mutagenesis. Mutation discovery is challenging as it requires a substantial amount of sequencing and analysis to detect very rare changes and distinguish them from noise. Also challenging are the cases when the organism of interest has not been sequenced or is highly divergent from the reference.

**Results:**

We describe the development of a simple method for reduced representation sequencing. Input DNA was digested with a single restriction enzyme and ligated to Y adapters modified to contain a sequence barcode and to provide a compatible overhang for ligation. We demonstrated the efficiency of this method at SNP discovery using rice and arabidopsis. To test its suitability for the discovery of very rare SNP, one control and three mutagenized rice individuals (1, 5 and 10 mM sodium azide) were used to prepare genomic libraries for Illumina sequencers by ligating barcoded adapters to *NlaIII *restriction sites. For genome-dependent discovery 15-30 million of 80 base reads per individual were aligned to the reference sequence achieving individual sequencing coverage from 7 to 15×. We identified high-confidence base changes by comparing sequences across individuals and identified instances consistent with mutations, i.e. changes that were found in a single treated individual and were solely GC to AT transitions. For genome-independent discovery 70-mers were extracted from the sequence of the control individual and single-copy sequence was identified by comparing the 70-mers across samples to evaluate copy number and variation. This *de novo *"genome" was used to align the reads and identify mutations as above. Covering approximately 1/5 of the 380 Mb genome of rice we detected mutation densities ranging from 0.6 to 4 per Mb of diploid DNA depending on the mutagenic treatment.

**Conclusions:**

The combination of a simple and cost-effective library construction method, with Illumina sequencing, and the use of a bioinformatic pipeline allows practical SNP discovery regardless of whether a genomic reference is available.

## Background

Mutations caused by base changes can occur spontaneously during mitosis or meiosis, or through alterations of mechanisms required for fidelity of replication and repair, or through exposure to mutagenic environments. Measuring the mutation rate is important for evolution, biochemistry, medicine and functional genomics. We are specifically interested in the functional genomic tool called TILLING (Targeting of Induced Local Lesions IN Genomes) [1]. The combination of efficient mutation discovery via high-throughput sequencing and the ability to generate allelic series (missense, nonsense mutations) enables reverse genetics in many species with limited genomics resources. However, populations with optimal mutation densities are necessary for screening efficiency and optimizing mutagenic treatments requires measuring mutation densities. For this purpose, PCR amplicons representing selected loci can be screened for mutations by mismatch-detecting assays [1] or by high throughput sequencing [2,3]. Both approaches, however, require testing of several hundred individuals [1]. With the advances in sequencing throughput, the entire genome of an individual can be resequenced with sufficient coverage to call changes with high reliability [4], or sequencing can be targeted to the exome by capture with complementary oligonucleotides [5]. Both methods, however, are still relatively expensive or laborious and required prior knowledge of the genome of interest or development of an oligonucleotide set suitable for exome capture.

A convenient approach to reduce genomic complexity for shotgun sequencing involves phasing the sequencing entry points at restriction enzyme sites to provide increased coverage of a subset of DNA regions [6]. Judicious selection of restriction enzyme and fragment size range can allow a coverage range that maximizes both discovery and economy [7,8], even for large genomes. Our variation of this method, RESCAN (Restriction Enzyme Sequence Comparative ANalysis), involves simple Illumina library construction using as little as 100 ng of input DNA and can be multiplexed (≤ 96 individuals) by employing custom barcoded adapters. The method allows genotyping using both the entry point restriction enzyme site and the adjacent sequenced DNA. If a reference sequence is not available, RESCAN read populations are intrinsically simpler than those derived from random fragmentation sequencing libraries and should be amenable to the construction of a reduced reference genome. Here, we describe development of this method and its application for discovery and detection of Single Nucleotide Polymorphisms (SNP) induced by mutagenesis, a type of variation much rarer and thus more difficult to detect than natural SNP. We demonstrate its capabilities in the characterization of mutation density in single individuals with or without the use of a reference genome. The method greatly facilitates the development of optimally mutagenized populations.

## Results

### Method development

**Figure 1:**
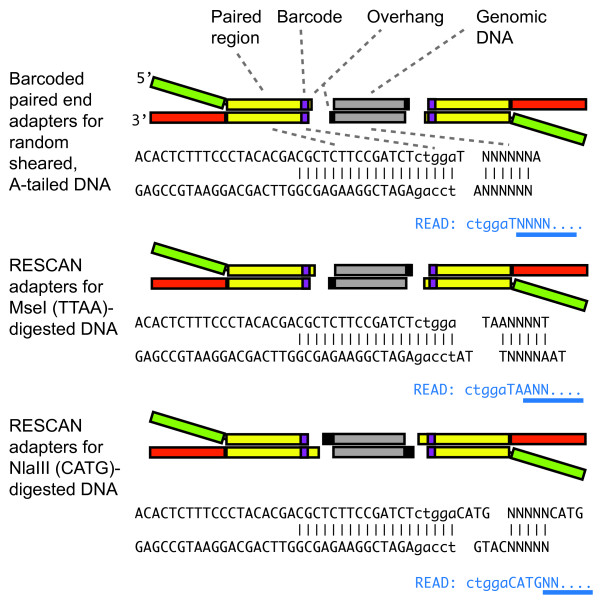
**Structure of barcoded adapters used for RESCAN**. The RESCAN adapters leverage the Y-adapter system used for standard Illumina sequencing libraries in which random-sheared, A-tailed insert DNA (grey boxed regions or NNN) is ligated to T-overhang formed by the paired adapters (top). The Y-adapter is formed by two oligonucleotides. A sequence barcode (lower case) is included adjacent to the end. For ligation to restriction enzyme-formed overhangs, the required extension is incorporated in the appropriate oligonucleotide of the adapter. Below each paired adapter sequence the beginning of the resulting sequence read is shown in blue, with the nucleotides that are not fixed, i.e. not part of the adapter, barcode and overhang, underlined in blue. The barcode length used in the early method-refining part of this work was of four bases. Five bases is the preferred length at the time of writing this paper because the first five cycles of Illumina HiSeq platform require random and similarly weighted base composition.

**Figure 2:**
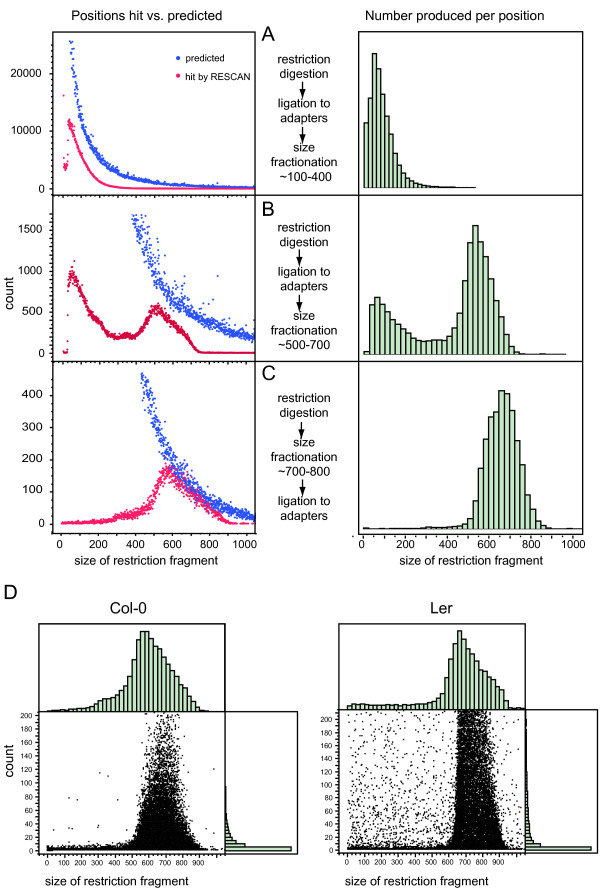
**Size distribution of RESCAN is affected by library construction and genotype**. The size of the restriction fragment sequenced in the RESCAN was calculated from the aligned reference genome. A, B, C. Effect of the method used for the construction of the library on the sampling of fragments. In the left graphs, the blue and red datapoints report respectively the number of total restriction fragment ends available in the genome for the indicated size (before fractionation) and the number sampled by one or more RESCAN reads. The blue points represent the same distribution in A, B and C, but zoomed on different Y-axis values. The right graphs report the distribution of number of RESCAN reads by size. All size fractionation in these preliminary experiments was done by gel electrophoresis and extraction of DNA from a selected section of the gel. D. Effect of a divergent genotype on the range of fragments sizes. The sequencing libraries for *A. thaliana *Col-0, the accession from which the reference genome is derived, and Ler, a divergent accession, were prepared according to protocol in C. The count of each RESCAN read is plotted versus the reference-deduced size of the restriction fragment to which it mapped. Many high coverage RESCAN reads from the Ler genome occur for fragments whose sizes (according to the Col-0 reference sequence) are not in the correct coverage size range. These cases are assumed to correspond to restriction size polymorphisms.

**Figure 3:**
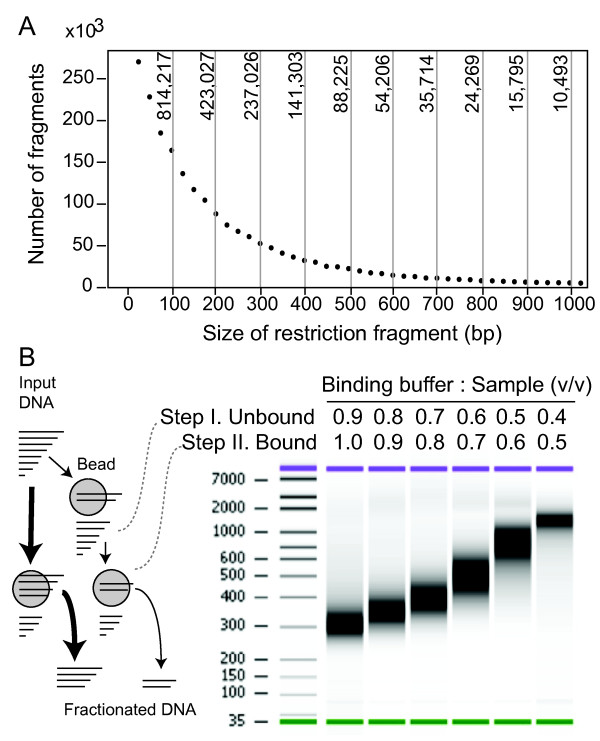
**Size fractionation of digested DNA by affinity beads**. A. Counts of restriction fragments by size after in silico digestion of the *Oryza sativa *Os6.1 genome with *NlaIII*. The Y-axis of the graph displays the count per 25 bp bins. The graph top axis displays the total count for in silico slices of 100 bp. The graph demonstrates how a size fraction from 100 to 200 bp would contain more than ten times the number of fragments found in the 600 to 700 bp fraction. B. Fractionation strategies with SPRI magnetic beads. On the left, a bottom-delimited size fraction of the digested input DNA can be taken in a single step (thicker arrows path), or a sliced size fraction in two steps (thinner arrows path). Slicing is demonstrated in a digital electrophoretogram on the right. In practice, bottom delimiting in a single step is the most practical solution since the larger size fragments contribute relatively less to the final library.

We devised a method (RESCAN) for the simple production of restriction enzyme-phased libraries for Illumina sequencers. The method entails digestion of the input DNA with a restriction enzyme, optional selection of a size range, ligation to modified Illumina Y adapters that feature sticky ends complementary to those produced by the enzyme (Figure 1), clean up of the ligation product, enrichment by PCR, and finally sequencing. To optimize this method, we used input genomic DNA from rice and arabidopsis, two model systems with well-characterized genomes [9,10]. Figure 2 compares the effect of the order of size selection versus ligation to the adapters by comparing the yield and size of sequenced fragments to the total number predicted from the genome sequence of the target. The importance of choosing the right molecular weight fraction is further demonstrated in Figure 3. The libraries were processed as described in the Methods and sequenced in Illumina GA.

The reads were aligned to the reference genome with Eland (Illumina, Inc). The position at which each read initiated was then extracted and the size of each corresponding restriction fragment was tabulated. The distribution of observed hits is displayed in Figure 2 for each library construction strategy and compared to the corresponding genomic total. Each library generated a nearly-normal distribution centered within the targeted range. Ligation followed by fractionation, however (Figure 2-A, B), produced a bimodal curve with a maximum corresponding to low molecular weight fragments and another maximum corresponding to the selected range. Contamination by the small fragments could be minimized by pre-selection of the target size range followed by ligation (Figure 2-C).

Restriction fragment length polymorphisms are expected in different accessions and may shift a fragment into or outside of the optimally covered size range (Figure 2-A, B, C). The former instances should be manifest as outliers in a graph of coverage by size. This expectation was verified in the accession Ler (Figure 2-D) where frequent fragments with good coverage are observed outside the optimal size range.

Some RESCAN reads mapped on the reference genome appeared not to start at an *MseI *site, but at sites differing by one base that we called proto sites. These degenerate sites may be cut by restriction enzyme star activity or they can be diagnostic of a polymorphisms between the reference genome and the sample [11]. In the case of MseI fragments, modifications of the fourth base (incidence of TTAB vs TTAA sites, where B is a base other than A), can be identified because the sequence read from these sites would start with, respectively, "...TTABNNN..." vs "...TTAANNN..." (Figure 1). We measured the frequency of star cutting by counting the two read types. The incidence of TTAB sites in 2.6 M reads was 0.22% indicating that star activity was very low.

**Figure 4:**
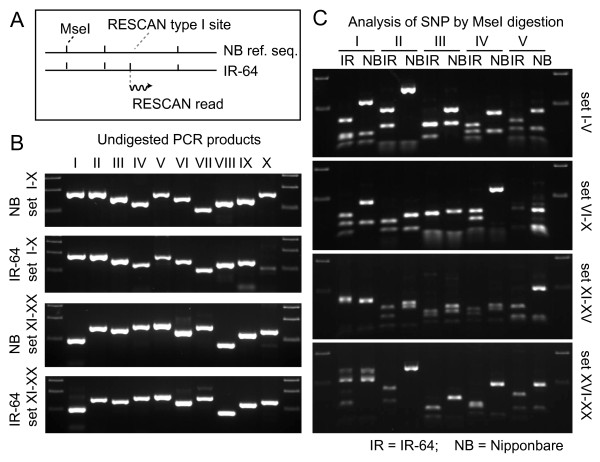
**Confirmation of SNP detected by the RESCAN type I approach**. A. RESCAN type I SNP can be identified in sites that are found for the target restriction site in the query (in this case IR64) but are absent in the reference. In most cases, examination of the reference sequence reveals the presence of a proto sequence, i.e. a sequence that diverges by one base from the expected sequence TTAA: VTAA, TVAA, TTBA, TTAB, where V and B are, respectively, not T and not A. For a proto such as GTAA, a T > G SNP is inferred. A SNP cannot be inferred for a proto site such as TTTAG since either T3 > A or G5 > A could have produced the *MseI *site. B. We chose 20 type I sites that allowed inference and were detected through 1 or 2 RESCAN reads. The products amplified using flanking PCR primers from Nipponbare and IR64 are shown. C. The amplified products were subjected to digestion with *MseI *and analyzed by agarose gel electrophoresis. The presence of an extra restriction site in the amplified IR64 DNA and not in the control Nipponbare is evident in 17 of the 19 amplified products, confirming the presence of a SNP producing a restriction site in IR64.

Reads initiating at reference sequence proto sites should increase with phylogenetic distance. RESCAN reads from the reference variety Nipponbare, two California varieties of japonica rice, and two indica varieties displayed progressively higher proto read counts (Table 1). These reads were highly predictive of polymorphism even at very low coverage: 17/19 proto sites tested were digested by *MseI *in IR64 and not in Nipponbare, confirming an *MseI*-associated polymorphisms (Figure 4). Depending on the proto site context, the polymorphism could be called unequivocally (see Methods). The inferred SNP corresponding to the proto to full site conversion were called Type I contrasting to the Type II SNP, detected within the reads. For example, with TTAB (where B = T, G or C) proto sites, 1200 out of 3000 predicted B > A SNP confirmed SNP already present in the NCBI rice SNP database.

**Table 1:** SNP discovery in rice from type I RESCAN

Accession	Type	Total	Off site	Proto	%
Nipponbare	Japonica	273,959	566	400	0.15

M-206	Japonica	156,710	1543	1100	0.71

M-202	Japonica	375,081	4483	3194	0.86

IR64	Indica	2,598,754	55024	37905	3.31

IR50	Indica	264,618	11450	8490	3.35

Gel-based electrophoretic fractionation (size selection) is cumbersome and not easy to scale up. We substituted it with Solid Phase Reversible Immobilization (SPRI) on magnetic beads [12,13]. Clean up of digested DNA with SPRI beads removed the bulk of the small restriction enzyme fragments. Different size cuts could also be carried out because the molecular weight of the bound DNA could be changed by the strength of the binding buffer (see Figure 3 and Methods). The method was tested and worked well with another restriction enzyme, *NlaIII*. The ratio of adapters to input DNA required careful adjustment to avoid adapter dimers. The optimal ratio was considerably lower than that used for regular Illumina libraries [12]. The resulting protocol proved robust and scalable as we increased the number of barcoded adapters from the few used above to as many as 96. The amount of input DNA digested with either 4 bp cutter enzyme could be as little as 100 ng without loss in efficiency.

### Discovery of rare polymorphisms

The method described above proved efficient at genotyping individuals in populations (Monson-Miller et al., unpublished results). A more challenging type of variation is the one resulting from mutagenesis because induced mutations occur at density lower than natural polymorphisms. For example, a well mutagenized population of rice has one base change every 250 kb of diploid DNA [14], which is about thousand time less frequent than the natural SNP density between japonica and indica rice [15].

**Figure 5:**
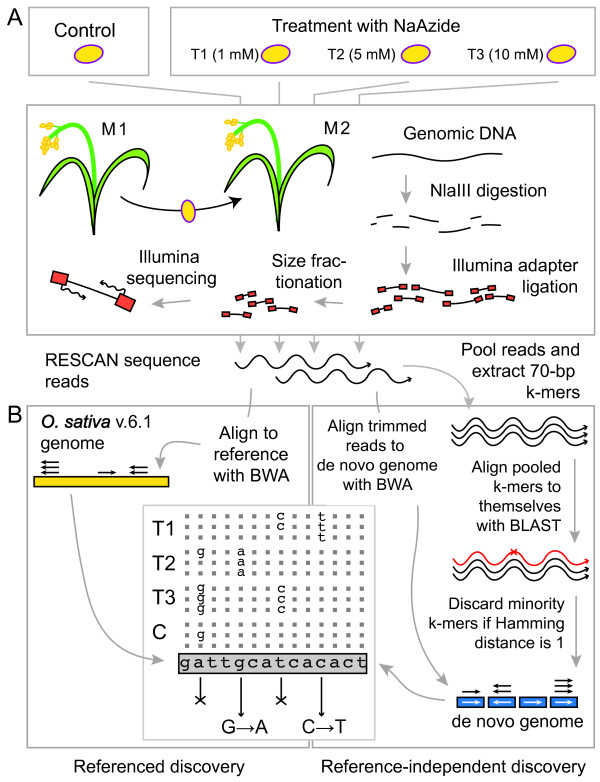
**Overview of experimental material and mutation discovery strategy**. The figure summarizes the steps undertaken in the mutation analysis. A. Plant mutagenesis, growth of M2 plants and production of RESCAN libraries. B. Informatic strategy for identification of mutations. The panel compares the bioinformatic process used with the genomic reference (left) and without (right). The table in the center bottom illustrates the strategy to identify mutations, which are expected to occur both as heterozygous and homozygous changes. T1, T2, T3 are mutagenized individuals. C is a control. For each position, calls concordant with the reference are dots, those discordant are base symbols. In the case of the second base A > G changes are found in multiple individuals and therefore cannot represent mutations (cross-out symbol is used). The fifth base G, however, displays changes unique to a single mutagenized individual. The G > A change is accepted. BWA and BLAST refer to the alignment programs used.

To test the capabilities of the RESCAN system for discovery of induced mutation rates in plants we developed the experimental protocol described in Figure 5. We tested the effect of varying sodium azide (NaAzide) concentrations on mutation rate in rice (*Oryza sativa*) cv. Kitaake. Table 2 illustrates the progressively more deleterious effect of increasing NaAzide concentration. The highest treatment resulted in less than 2% survival to the M2 generation vs. ~20% for the next lower treatment. The genomic DNA of three M2 individuals derived from 1 mM (T1), 5 mM (T2) and 10 mM (T3) NaAzide treatments and of a single control individual (called "C") was used for RESCAN library preparation with the restriction enzyme *Nla*III. The four indexed libraries were each sequenced using 85b × 2 paired-end reads on one and one half lane of the Illumina GAII sequencer.

**Table 2:** Mutagenesis of rice by NaAzide

NaAzide	Survival to maturity		M1 Fertile (at least 1 seed)	
mM	count/total	%	count/total	%

0	133/150	88.7	133/133	100

1	301/398	75.6	299/301	99.3

5	152/400	38.0	82/152	53.9

10	16/400	4.0	7/16	43.8

#### Reference-dependent detection

**Figure 6:**
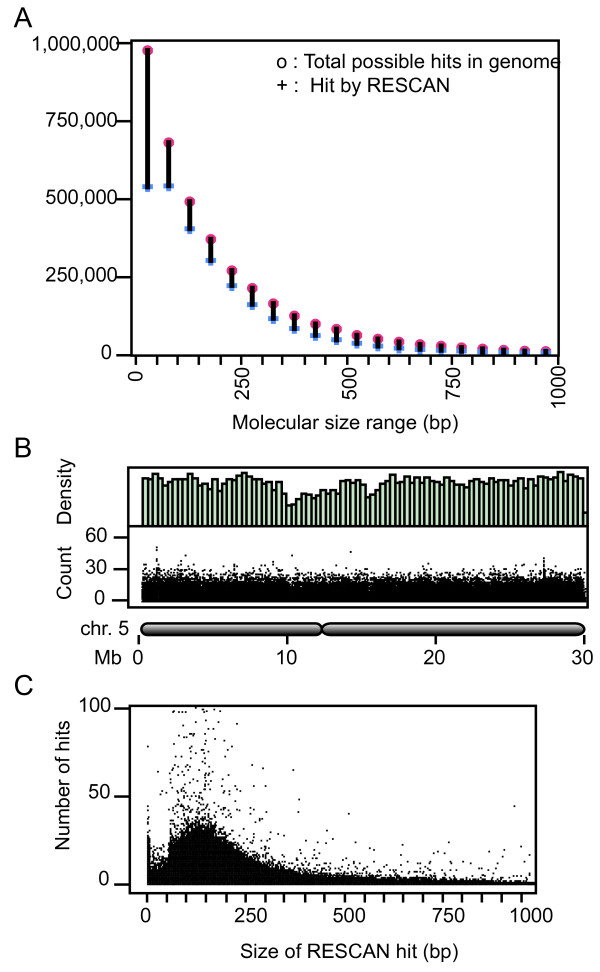
**Distribution of the RESCAN reads used for mutation discovery**. Different views of the distribution of the RESCAN reads derived from the control individual "C". A. The red and blue datapoints report respectively the number of total restriction fragment ends available in the genome for the indicated size range (before fractionation) and the number covered by one or more RESCAN. The bar joining the two points highlights the difference. B. Exemplary data for chromosome 5 of rice. The top histogram displays the density distribution of the forward RESCAN reads. The bottom graph plots the count for each RESCAN read vs. the position on chr. 5. The schematic drawing below the chart illustrates the position of the centromere on the chromosome. C. The graph plots read counts for each of the forward RESCAN positions vs the predicted size of the restriction fragment involved. The rescan library for individual T1 has similar properties. Those for individuals T2 and T3 have about double the total number of reads.

We used the published rice cv. Nipponbare genome [9,16] to align the RESCAN reads using the program BWA [17] with default settings. The analysis of expected and predicted restriction fragment sizes (Figure 3 and 6) demonstrates that the method using the SPRI bead-using method is comparable to size-selection after gel electrophoresis. By selecting fragments in the 100 to 250 bp size range we probed a relatively larger component of the genome (about 1/5). Using a custom parsing script, we searched for candidate SNPs in the resulting alignment. We filtered out SNPs corresponding to poorly mapped reads and those that occurred in repeated regions by setting a maximum cumulative allowable coverage of 200. We further required that candidates for homozygous mutations be unique to one individual and occur in sites where coverage was at least 2 in that individual and that all calls be identical and that position was covered at least once in each of the other 3 individuals (see Methods for details). For heterozygous mutations the general criteria were similar, but, expectedly, we encountered higher noise. Noise was evidenced by the high numbers of potential mutant calls in the control and in all genotypes by a high frequency of base change types that were not expected from the mutagenic action of NaAzide (see below). We found that using a minimum of 5 or more variant calls as a bottom threshold largely eliminated the noise.

#### Reference-independent detection

Assessment of mutation density can be difficult if a reference genome is unavailable or diverges too much from the query sequences. In a parallel experiment, the same raw sequence data were used for reference-independent SNP discovery. We used the first 70 bases of each read to construct a list of 70-mer substrings (termed k-mers). We curated this set by eliminating the k-mers that had higher-than-expected coverage (potential repeated regions) and by discarding the minority member in pairs that had a Hamming distance of 1 [18]. We then used the resulting k-mer set as a *de novo *reference. The first 65 bases of each RESCAN read were aligned to this reference using BWA with a maximum mismatch allowance of 1. The resulting alignment was parsed as for the reference genome alignment.

### SNP types are consistent with a chemical mutagenesis mechanism

**Figure 7:**
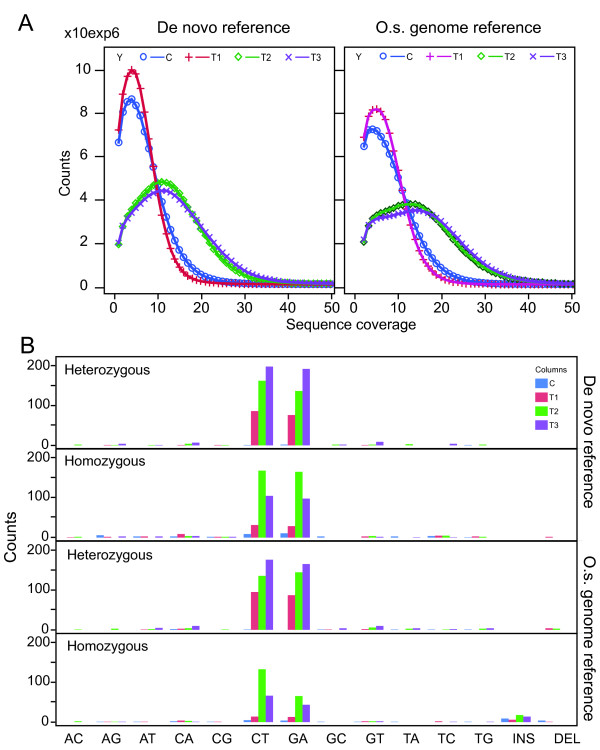
**Pattern of SNP frequency**. A. The graphs illustrate the relationship between counts and coverage for the surveyed positions in the four tested libraries. Approximately half the number of reads were obtained from the control (C) and T1 libraries as for the C2 and C3 (Table 3). B. The absolute SNP count is shown for the tested individuals, using four bars listed in the order (C, T1, T2, T3) for each change type. Mutagenized individuals (T1, T2, T3) display increased SNP types consistent with the mutagen action (GC > AT) while the untreated individual (the first bar of each group) displays only background changes in both de novo referenced and *O.s*. genome referenced analyses. Changes that differ statistically from the expectation of random sequencing errors are marked by the asterisk.

For each treatment, the *de novo *reference (Table 3) was about 5% larger than the genome-aligned space (about 82 to 87 vs 78 to 82 Mb, see Table 3 for details). In both cases a set of SNPs consistent with mutation sites were identified. Treatment with NaAzide is expected to yield only or predominantly GC > AT (same as G > A and C > T transitions) changes [19]. For the mutagenized individuals, GC > AT SNPs were more frequent than other SNP types in both types of analysis (Figure 7). Candidates appeared to be randomly distributed throughout the genome. The fraction of GC > AT changes observed in all T2 and T3 measurements (Figure 7) was significantly different from the expectation of random sequencing error [2]. For T1, only the heterozygous changes were significant. Fewer SNPs were identified using the *O. sativa *genomic reference than using the *de novo *reference: 347 vs 623 for putative homozygous mutations and 863 vs 813 for putative heterozygous mutations. Of the 347 GC > AT putative homozygous changes found with the *O. sativa *reference, 247 (71%) were shared with the *de novo*-referenced analysis. Of the other changes, only 21% were shared. For the heterozygous mutations, overall 75% of the positions present in the referenced analysis were also present in the *de novo*-referenced analysis. On average, the GC > AT base changes were confirmed much more often (80%) than the other types of changes (25%).

**Table 3:** Sequencing coverage

Reference	Read Alignment	Control	T1	T2	T3
None	Total number of reads (million)	15.5	13.7	28.5	29.9

*O.s*. 6.1^1^	Number of mapped reads (million)	13.4	11.8	24.6	26.4

*O.s*. 6.1	Complexity (Mb)	131.8	131.5	142.8	142.4

*O.s*. 6.1	Complexity, homozygous changes^2 ^(Mb)	78.2	77.7	82.7	82.6

D*e novo*^3^	Number of mapped reads (million)	13.3	11.7	24.5	26.3

D*e novo *	Complexity (Mb)	102.4	102.1	106.9	106.7

D*e novo *	Complexity, homozygous changes^2 ^(Mb)	82.6	82.1	87.4	87.3

^1^Reference complexity: 380 Mb. ^2^Complexity for homozygous scoring is calculated according to the following requirements: i) covered at least once in each of the four libraries, ii) covered at least twice in the putative mutant, iii) covered less than 200 times cumulatively in all libraries. ^3^Reference complexity: 117 Mb.

### Measurement of mutation rate

To determine the mutation rate, the count of unique SNPs in each treatment was divided by total number of bps effectively assayed (see Methods for details and Table 3). The number of putative homozygous SNPs per Mb in the *O. sativa *genome-referenced analysis was 0.4 for the control, 0.58 for T1, 2.7 for T2, and 1.6 for T3. For the *de novo*-referenced analysis the number was 0.54 for the control, 1.1 for T1, 4.07 for T2, and 2.53 for T3. These values were fairly similar suggesting that reference-less alignments can be as effective as reference guided ones for discovery of very rare polymorphisms. Deriving the total mutation rate can be complicated by the assumptions used and is expected to be sensitive to the coverage (see Discussion). Since the above calculation did not include the heterozygous SNP, it is an underestimate of the real mutation rate. Depending on the number of heterozygous mutations that were classified as homozygous (see Discussion) the actual mutation density may range from less than 3 times to 3 times the one reported above. Nonetheless, the estimated mutations densities were generally consistent with previous work [2] and provide a guide in the design of future mutagenesis experiments.

## Discussion

We demonstrate the use of a simple method to identify extremely rare SNP in the genome of individuals and estimate the connected mutation rate, independent of availability of a reference genome. The method takes advantage of the facile construction of libraries for Illumina sequencers using restriction-digested genomes. Construction of reduced representation libraries using restriction enzymes was first described for Sanger sequencing [6]. The method has since been applied to high throughput sequencing library construction for the 454 platform [20], and for Illumina [7,8,21-23]. These approaches have been reviewed recently [22]. Restriction site associated DNA tags (RAD) sequencing, described four years ago, has found multiple successful applications [22,24-31]. Our method differs from RAD sequencing because a genomic fragment in the sequencing library is defined by two symmetric restriction sites instead of asymmetric combination of a restriction site with a randomly fragmented and flushed end, thus being most similar to that of Andolfatto *et al. *[23] and of Elshire *et al. *[8]. The one-step ligation for library construction used by all three methods provides a simple, robust and easily implemented step. Our method differs from the first because the terminal portion of the Illumina adapter sequence is left intact allowing the use of the standard Illumina sequencing primers and combined sequencing with regular libraries. It differs from the second by the use of a single Y-adapter instead of two fully double-stranded ones and the placement of the same barcode on both sequencing reads derived from paired-end sequencing (Figure 1). Double barcoding identifies potential chimeric products in multiplexed libraries that are sequenced as paired reads. The method employs oligonucleotides with desalted purity and no phosphorothioate modification, enabling considerable savings in setting up 96-barcode multiplexing. Similarly to the two methods above, different types of overhangs can be used. When used with a two-base overhang producing enzyme such as *MseI *(T↓TAA), the adapter can be ligated to the insert in the presence of the restriction enzyme because adapter-insert ligation eliminates the restriction site. When used with a four-base overhang producing enzyme such as *NlaIII*, the restriction enzyme must be removed or inactivated.

The complexity of the sequencing library can be modified by the choice of restriction enzyme and by size fractionation of DNA. Six-base cutters reduce complexity compared to four-base cutters. For example, the six-base cutter *SphI *(GCATG↓C) can be used with the *NlaIII *(CATG↓) adapters achieving satisfactory reduction in complexity of large genomes (> 10 Gb; Monson-Miller and Comai, unpublished results). SPRI bead cleanup before and after ligation of the digested DNA can be tailored to produce different molecular size cuts. Although size selection can be achieved by excision of DNA from agarose gel after electrophoresis, we found that the latter method is less efficient, more laborious and not easily scalable. A drawback of the SPRI bead-based fractionation is that removal of the abundant smaller fragments can be incomplete, resulting in their capture through intrafragmental ligation and chimeric library products. These instances complicate analyses based on assumed contiguity of the paired end reads. Often, however, the two reads are queried independently and this is not a problem.

Analysis of reduced complexity libraries starts by mapping quality-filtered reads to a reference genome using software such as ELAND or BWA. BWA outputs two file types useful in this application: SAM files and pileup files. The first provides the entry point of each read identifying restriction sites common to the input and the reference and restriction sites unique to the input and not present in the reference. Interestingly, the latter sites can be highly predictive of SNP even with very low coverage (one or two reads, Figure 4). For example, using *MseI *(T↓TAA) we demonstrated that sites where the fourth base (TTAA) is verified in the read and the corresponding reference proto site is TTAB, are confirmed in over 80% of the tested cases (Figure 4). Of 3000 SNP inferred B > A SNP, 1200 were confirmed in the databases. We believe that the remaining SNP are likely to be real as well.

More commonly, SNP discovery employs the sequence of the read beyond the restriction site, a method that requires higher coverage, but is more productive because it queries more sequence (currently 100 bases vs the 4 of a restriction site) and can provide codominant information for any SNP discovered. Detection of these SNP is achieved by parsing the pileup table produced by BWA, where calls for each position are listed with the corresponding qualities [17]. We searched for changes induced by NaAzide, which compared to changes derived from natural variation represent a considerable challenge because they are much rarer than variation SNP [1,15]. Typical mutations are present in mutagenized diploid genomes with frequencies ranging from less than 0.5 to 10 per 10^6 ^bp of diploid DNA. Furthermore, while natural variation SNP are shared by multiple individuals and can thus be confirmed through biological replication, most mutations affect a single individual. We were able to detect induced changes by applying a common sense strategy (Figure 5, see methods). We were helped by the specificity of the induced changes: the mutations conformed the NaAzide mutagenic spectrum detected in barley and consisted almost exclusively of G:C to A:T transitions [19] in contrast to the 28% G:C to A:T (accompanied by 28% A:T to G:C) transitions expected from natural variation [32].

Reference-independent discovery using k-mers found more SNP, including 70-80% of those found in the *O. sativa*-referenced discovery. The fact that 20 to 30% of the potential mutations found in the reference-analysis were not found in the k-mer analysis can partially be explained by the fact that the k-mers were trimmed (from 75 bps to 70 bps) and the reads were further trimmed (from 75 bps to 65 bps, resulting in an effective loss of 14% of the sequence information). Another factor differentiating the two approaches is the potentially different treatment of repeated regions. SNP that are in known repetitive regions would be excluded in the referenced search. However, if only one of the repeat was represented in the RESCAN library, it would behave as single copy and be scored in the reference-independent discovery. Such cases may contribute to the efficiency of *de novo*-referenced discovery.

A considerable challenge in the analysis is constituted by the presence of heterozygous mutations, which in M2s are expected to be 2/3 of the mutant sites. One difficulty lays in distinguishing homozygous from heterozygous sites: for example, a base position for which three calls are all variant could be homozygous mutant or heterozygous with associated probabilities of 0.875 vs 0.125 (0.5^3^), respectively. Similarly, an heterozygous site could yield three wild-type calls with a 0.125 probability. A second difficulty, connected to the first, lies in estimating the covered genome because each coverage level has an associate probability of detection. In order to reduce noise, we set our algorithm to call heterozygous sites only if they carry a minimum of 5 mutant calls. For example, if a 100b DNA was sequenced to a coverage of ten, any heterozygous site would yield call ratios according to the binomial distribution resulting in a connected probability of detection of 0.62 (X ≥ 5, p = 0.5). Because heterozygotes are detected with lower efficiency, estimating the mutation density under these conditions would require adjusting the number of bases effectively assayed to 62 bases instead of 100. In practical terms, this is laborious and may require the careful construction of an adequate statistical model. A simpler solution to the heterozygous problem would be increasing the coverage, which can be achieved as discussed above. For the purpose of our estimate, we derived a mutation rate using a simplified calculation with the homozygous mutants. We estimate that this might be between half and one third the real mutation rate, depending on the fraction of putative homozygous sites that are actually heterozygous.

Number of SNPs consistent with NaAzide mutagenesis was higher in all 3 treated individuals than in the control, and mutation density peaked in the intermediate treatment according to the homozygous calls. This is not the case when considering the heterozygous calls. If we consider the homozygous analysis to be more accurate, a plausible outcome, this behavior requires potential explanations. The lethality of the mutagenic treatments increased from low to a very high 96% in the 10 mM NaAzide treatment. It is possible that the severity of the 10 mM treatment may be counterproductive and that, for example, survivors may have escaped the full treatment. A similar observation has been reported in barley using sterility as a proxy for mutation density [33]. Alternatively, variation may result from the limited sampling. It is possible, for example, that different cell types in the embryo may respond differently to the mutagen and subsequently enter stochastically the transient germ line that gives rise to plant gametes [1]. Therefore, the extent of individual variability remains to be assessed.

The method described here should be applicable to more studies than just those focusing on mutagenesis. For example, it will allow mapping and backcrossing of induced mutations in the background of the same accession used for mutagenesis by providing markers that allow discrimination of the mutagenized genome from the wild type. It should also allow comparison of substrains of the same variety and, if sufficient SNP are found, genetic characterization of diverged traits. We have also applied the RESCAN to natural SNP discovery and mapping in rice (Tai *et al*., unpublished results), *Arabidopsis suecica *(Henry and Comai, unpublished results) and *Arabidopsis thaliana *[34]. In all these systems, RESCAN proved robust in its application and analysis.

## Conclusions

We describe here the development and application of a simple and economical method for reduced representation sequencing. We demonstrated it effectiveness by measuring the mutation rate in multiple individuals. RESCAN libraries, made by direct annealing and ligation of adapters to digested fragments of genomic DNA, are easy to multiplex and analyze. Coupled to the ability to assay a genome without a reference, the method should facilitate genotyping as well as the measurement of mutation densities in many systems.

## Methods

### Mutagenesis with sodium azide

Seeds of *O. sativa *ssp. *japonica *(cv. Kitaake), a variety closely related to the reference cv. Nipponbare, were pre-soaked in ultrapure water for 20 hours at 25°C prior to sodium azide treatment. For mutagenesis, sodium azide solutions of 1 mM, 5 mM, and 10 mM were made in 0.1 M sodium phosphate buffer, pH 3. Batches of 100 seeds were treated with 27 ml of sodium azide solution in a 50 ml tube at RT (22-24°C) for 3 hours. Sodium azide was decanted and seeds were washed 3 times with 30 ml of ultrapure water for 5 minutes each time. Seeds were then transferred to germination paper in a standard plastic petri dish for germination at 25°C. Control Kitaake seeds (neither presoaked nor treated with sodium azide) were plated at the same time. After 7 days, germinated seeds were transplanted to UC soil mix C and grown in greenhouse to produce M2 seeds. M2 seeds were planted directly in UC soil mix C and leaf tissue was harvested for DNA isolation.

### DNA extraction

Total genomic DNA was isolated from frozen leaf tissues that were mechanically ground prior to extraction using a potassium acetate-SDS method [35].

### RESCAN library construction

#### Method development

Approximately 1000 ng of DNA was digested with the restriction enzyme *MseI *(T↓TAA, NEB, Ipswich, Massachusetts, cat. no. R0525) for 1 to 6 hour at 37°C. After the completion of digestion was verified by agarose gel electrophoresis, the DNA was purified with a Qiaquick PCR purification minicolumn (Qiagen, Germantown, Maryland, cat. no. 28104) and resuspended in 40 μl of 10 mM Tris buffer. Alternatively, the desired size range of genomic fragments was excised from agarose gel after electrophoretic separation, extracted with a Qiaquick gel extraction kit (cat. no. 28704) and resuspensed in 20 μl of 10 mM Tris buffer. For ligation, 20 μl of genomic DNA, either from the total digestion, or the size cut, were combined in a final volume of 44 μl with T4 DNA ligase (various manufacturers), the ligase buffer provided by the manufacturer, 0.5 μl of T4 DNA ligase, 0.5 μl of *MseI *(except for agct barcode, see below), and 1 μl of 0.05 μM premixed adapter oligonucleotides to form the single end sequencing Illumina Y adapter. The use of *MseI *during ligation depended on the sequence of the adapter and was employed whenever possible to minimize ligation between genomic fragments. The sequence of the adapter oligonucleotides is shown below, with the barcode in lower case. Additional barcodes used were (shown as the adA2 oligonucleotide strand sequence): gata, cacc, tagc, agct, ctag. Note that all, except "agct", cause loss of the *MseI *site upon ligation to an *MseI *fragment. The adapter sequences are shown for the record, but are no longer suited for the 2012 and later Illumina sequencing platform. The oligonucleotides, prepared at desalted quality, were obtained from Life Technologies (http://www.invitrogen.com).

adA2_GGTG: P-TAggtgAGATCGGAAGAGCTCGTATGCCGTCTTCTGCTTG

adB2_GGTG: ACACTCTTTCCCTACACGACGCTCTTCCGATCTcacc

Ligated DNA was purified on a Qiaquick column, enriched by PCR amplification with Illumina PCR amplification primers for 16 to 18 cycles and examined by analytical gel electrophoresis. A slightly diffuse band in the target range of molecular weight (insert size + ~100b of adapters) was diagnostic of the desired outcome. The presence of excessive adapter dimer (a fragment in the 100-150 bp) was undesirable. If found, it could be removed by preparative gel electrophoresis, or it could minimized by repeating the procedure from the ligation step using a lower concentration of adapters.

Illumina primers:

pr1: AATGATACGGCGACCACCGAGATCTACACTCTTTCCCTACACGACGCTCTTCCGATCT

pr2: CAAGCAGAAGACGGCATACGAGCTCTTCCGATCT

Sequencing of the RESCAN libraries started with 25b reads on the original (2007) Genome Analyzer and progressively employed the improvements in chemistry and apparatus.

#### Standard method

Approximately 500 ng of DNA was digested with the restriction enzyme *NlaIII *(NEB, Ipswich, Massachusetts, cat. no. R0125) for 1 to 6 hour at 37°C. After the completion of digestion was verified by agarose gel electrophoresis, the DNA was purified selecting the desired molecular weight range. For this purpose AMPure SPRI Beads (Beckman Coulter Genomics, Danvers, Massachusetts, cat. no. A50850) were added in the suitable ratio to DNA (see Figure 3) and used to manufacturer's instructions. For example, to remove fragments smaller than 100 b the recommended ratio (1.8:1) was used. To remove fragments higher than a certain amount (top cut) SPRI beads in the desired ratio were applied and the unbound fraction was saved. Because the bulk of the fragments are always found in the bottom fraction (Figure 3), we commonly used conditions (i.e. ratios) that would remove all fragments below a certain molecular weight and directly proceeded with ligation of the remaining fragments to the adapter. Although this represented a range of sizes, the higher frequency of smaller fragments and their advantage during the amplification steps resulted in a de facto enrichment of the near-bottom class of fragments. This simplification shortened the protocol and made the method cheaper, important for high level multiplexing. Oligonucleotides for the barcoded Illumina adapters used in the mutation detection experiment were as follows (barcode in lower case):

Control, adA: P-atcacAGATCGGAAGAGCGGTTCAGCAGGAATGCCGAG

adB: ACACTCTTTCCCTACACGACGCTCTTCCGATCTgtgatCATG

The oligonucleotides were ordered as in desalted quality and were not modified except for phosphorylation of adA. The same oligonucleotides were used for control, T1, T2 and T3 samples, but for the barcode (respectively: atcac, ctctc, cgaat, and gagca). The oligonucleotides were mixed in a 1:1 ratio to form the adapter, which was stored and used as needed without any annealing pre-incubation. One μl of a 0.05 μM dilution of the adapter was added to the SPRI bead-fractionated DNA in a 44 μl ligation reaction employing the Quick Ligation Kit (NEB, cat. no. M2200). After 15 minute incubation at room temperature, the ligation reaction was cleaned with AMPure SPRI Beads, utilizing a 0.8:1 v/v ("bead in binding buffer":sample) ratio to remove smaller fragments (less than 250-bp) and unligated adapters. The libraries were enriched using a mix of 10 μl of template, 15 μl of Phusion 2x HF Master Mix (NEB, cat. no. F531), 1 μl of 5 μM premixed paired-end Illumina primers and 4 μl of water and the following amplification protocol: 30 sec at 98°C; 14 cycles of 10 sec at 98°, 30 sec at 65°, and 30 sec at 72°; and a final extension with 5 min at 72°. PCR product was purified using AMPure SPRI Beads with a 0.8:1 v/v (bead in buffer:sample) ratio. Libraries were quantified using the Agilent 2100 Bioanalyzer, and were sequenced according to manufacturer's instructions on one and one half lanes (3/8 lane per individual) of the Illumina GAII (Illumina, San Diego, California) with 85-bp paired-end reads.

### Computational analysis

Analysis during method development employed the Illumina alignment pipeline using the Eland program with the relevant updates of year 2007, 2008 and 2009. For the mutation analysis, the Illumina 1.5+ format (fastq) reads were filtered using a custom informatic pipeline (http://tinyurl.com/barcode-tool) that divided them based on barcode. Additionally, it removed the barcode sequences, adapter and primer sequences, reads shorter than 25-bp, and reads containing bases with Phred quality scores less than 20. Quality scores were converted to Sanger scale, which is compatible with most alignment programs.

**Mutation detection**: For referenced discovery: BWA (http://bio-bwa.sourceforge.net/) was used to align reads to the reference (Os 6.1, http://rice.plantbiology.msu.edu/) [16] genome with default mismatch allowance, producing an mpileup file with Samtools [36] (http://samtools.sourceforge.net/). The mpileup file contains the base calls at each position, for each library. Basecalls can be A, T, C, G, * (deleted base) or insertions (+AAT for example). This file was parsed in the following manner. First, any basecall with a sequence quality lower than 20 or a read with a mapping quality < 20 were discarded. Next, the basecalls for the four libraries were pooled and only positions that were collectively covered not more than 200 times were retained (to avoid repeated sequences). Positions that were not covered at least once in each of the four libraries were further discarded. If all basecalls were the same, that position was classified as homozygous and further classified as "ref" if the basecall was the same as in the reference genome or "SNP" if it was not. If there were more than 1 basecall, the following criteria were applied: If the least frequent basecall was found in more than 1 library and accounted for > 10% of the basecalls, the base was called heterozygous. If the least frequent basecall was found in more than 1 library and accounted for < 10% of the basecalls, the base was called homozygous.

This latter subset of positions was further assayed for the presence of potential mutations. Potential mutations were detected as follows: i) if there were only 2 different basecall (one dominant "WT" basecall and an another): if the non-WT base was observed at least twice from a single library and never from the other three, and there were no other basecalls for that library, the mutation was classified as potential homozygous mutation. If the non-WT base was observed at least 5 times but there were other basecalls for that library, it was classified as a potential heterozygous mutation. ii) positions for which more than two different basecalls were observed were dealt with in the following manner: If the least frequent basecalls were each only found once, the position was ignored for mutation detection purposes. Similarly, if the least frequent basecall was only found once, it was ignored and that base was processed as if there were only 2 basecalls (see above). If all basecalls were each found at least twice, that base was classified as "ambiguous" and removed from further analysis. This analysis was performed on reads aligned to the reference genome and reads aligned to the pseudo-reference.

The number of positions obtained in each of the categories described above are summarized as follows for *O. sativa*-based reference and for *de novo *reference, respectively: million bases in pileup table = 118.6, 101.9; total covererage > 200 and covered in all four samples (Mbases) = 84.7, 89.2; % SNP (as defined above) = 0.028, 0.0004; % heterozygotes = 0.019, 0.036.

To assess mutation rates, the number of putative mutations was divided by the number of assayed bases for each library. For homozygous scoring, these had to meet the following requirements: i) covered at least once in each of the four libraries, ii) covered at least twice in the putative mutant, iii) covered less than 200 times cumulatively in all libraries. For heterozygous scoring, the requirements were as follows: i) covered at least one in each of the four libraries, ii) covered less than 200 times cumulatively in all libraries and iii) mutant allele covered at least five times in the mutant library and never in the other three libraries. Positions covered less than 15 times were adjusted for random sampling effects of non mutant bases in a heterozygous background (see Discussion).

In order to determine how many potential mutations were found in both types of analysis, the k-mers containing potential mutations were aligned to the reference genome using BWA (as described above). The position of the potential mutation in the reference genome was extracted from the alignment and the position of the mutation in the k-mer. This set of positions were compared to the set of positions obtained from the referenced-analysis.

### Statistical tests

The Fisher Exact test was used to compare observed and expected (from sequencing errors) SNP type ratios. We derived an expected fraction of GC > AT in sequencing errors of 0.59 based on Figure 2c of Tsai *et al*.[2]

### Data, software and further information

Sequence reads used for the mutation analysis are available at NCBI Sequence Read Archive with the following accession number: [Sequence Read Archive:SRA049884.2]. Software and additional information on the RESCAN method are available at http://comailab.genomecenter.ucdavis.edu/index.php/RESCAN.

## Competing interests

The authors declare that they have no competing interests.

## Authors' contributions

LC, JMM and JF carried out preliminary method development, THT and LC conceived the mutation search project, THT supervised the mutagenesis and initiated the mutation search project, DCSM performed the mutagenesis and prepared the sequencing libraries; LC and IMH supervised the bioinformatic analyses. JMM and IMH performed the bioinformatic analyses; JMM, IMH, THT and LC wrote the manuscript. All authors read and approved the final manuscript.
